# An Optimal Enhanced Kalman Filter for a ZUPT-Aided Pedestrian Positioning Coupling Model

**DOI:** 10.3390/s18051404

**Published:** 2018-05-02

**Authors:** Qigao Fan, Hai Zhang, Yan Sun, Yixin Zhu, Xiangpeng Zhuang, Jie Jia, Pengsong Zhang

**Affiliations:** College of Internet of Things Engineering, Jiangnan University, Wuxi 214000, China; qgfan@jiangnan.edu.cn (Q.F.); 6171920010@stu.jiangnan.edu.cn (Y.S.); YixinZhu1987@jiangnan.edu.cn (Y.Z.); 6161920011@vip.jiangnan.edu.cn (X.Z.); 6171920006@stu.jiangnan.edu.cn (J.J.); 1070114134@vip.jiangnan.edu.cn (P.Z.)

**Keywords:** pedestrian positioning, attitude fusion filter, zero velocity update algorithm, OEKF

## Abstract

Aimed at overcoming the problems of cumulative errors and low positioning accuracy in single Inertial Navigation Systems (INS), an Optimal Enhanced Kalman Filter (OEKF) is proposed in this paper to achieve accurate positioning of pedestrians within an enclosed environment. Firstly, the errors of the inertial sensors are analyzed, modeled, and reconstructed. Secondly, the cumulative errors in attitude and velocity are corrected using the attitude fusion filtering algorithm and Zero Velocity Update algorithm (ZUPT), respectively. Then, the OEKF algorithm is described in detail. Finally, a pedestrian indoor positioning experimental platform is established to verify the performance of the proposed positioning system. Experimental results show that the accuracy of the pedestrian indoor positioning system can reach 0.243 m, giving it a high practical value.

## 1. Introduction

An indoor pedestrian positioning system is a system for real-time access to pedestrian location information in an enclosed environment [1]. The widely used Global Positioning System (GPS) can obtain highly precise positioning information outdoors. However, within enclosed environments, the satellite signal is easily disturbed by the building, and the GPS fails to provide accurate pedestrian positioning information [2]. At present, indoor positioning technology is roughly categorized into wireless and inertial positioning technology. Wireless positioning technologies include infrared [3], ultrasonic [4], Bluetooth [5], Wi-Fi [6], ZigBee [7], Radio Frequency Identification (RFID) [8], Ultra Wideband (UWB) [9], visual [10], and wireless network positioning technology [11]. These positioning technologies are affected by external factors such as non-line-of-sight factors and multipath factors [12]. Therefore, the accuracy of wireless positioning is not high enough and the stability is poor. Inertial positioning technology [13] obtains pedestrian velocity, position, and attitude based on an accelerometer and a gyroscope. The errors of inertial navigation are unaffected by the external environment, but the inertial navigation system is prone to cumulative errors over an extended period of time. Inertial navigation systems based on Microelectromechanical-Inertial Measurement Units (MEMS-IMU), which has advantages in terms of price, structure, volume, and weight, have drawn much attention in recent years [14].

A gait detection mode [15,16,17,18,19] and adaptive filter [20,21,22] have been designed to study the regularity of pedestrian kinematics and walking gaits to offset positioning errors. Integrated positioning systems are introduced to offset errors [23], including IMU/UWB [24], IMU/WSN (Wireless Sensor Networks) [25], INS/WIFI [26], and INS/RFID [27]. When the pedestrian is in an unknown enclosed environment, some integrated positioning systems will be ineffective. If the pedestrian is running or jumping, the errors in the pedestrian attitude cannot be effectively offset and, in turn, affect the accuracy of the velocity and position measurements [28].

In the filtering algorithm of the inertial navigation system, a two-stage filter is designed to effectively reduce the cumulative errors [29]. In practical applications, the characteristics of the noise in the positioning system which affect the positioning accuracy cannot be determined. Adaptive filtering algorithms have been adopted to reduce the drifts and errors, including the fuzzy logic adaptive filter [30], Sage–Husa Adaptive Filter (SHAF) [31], and Strong Tracking Filter (STF) [32]. The SHAF can estimate the statistical characteristics of noise in real time, but cannot identify outliers within the measurement data; this reduces the fault tolerance of the positioning systems.

To detect the outliers within the measurement data, least squares estimation, time polynomial extrapolation, and differential algorithms are introduced. However, these algorithms are vulnerable to false positives, false negatives, and delays. The threshold-based wavelet denoising algorithm is designed to detect outliers [33]. Aimed at the outliers in the dynamic measurement process, a self-adaptive five-point linear prediction data detection method was introduced, in which only the data of a single measurement can be selected and the error of slow change in the system cannot be effectively identified [34]. An anti-outlier filter based on orthogonality of innovation was used [35] to eliminate outliers and track the moving targets effectively. An Optimal Enhanced Kalman Filter (OEKF) algorithm, based on the simplified Sage–Husa adaptive filtering algorithm and the anti-outlier filter, is proposed in this paper. In the filter, orthogonality of innovation is used to detect outliers, covariance matching is adopted to judge divergence of filtering, and the activation function is taken to weight the measurement vector. This paper is organized as follows: Section 2 begins by modeling, analyzing, and reconstructing the errors of the inertial sensors using wavelet variance and the wavelet decomposition algorithm. Section 2 continues by correcting the cumulative errors in attitude and velocity using the attitude fusion filtering algorithm and Zero Velocity Update algorithm (ZUPT), respectively, and finishes by developing the proposed OEKF algorithm. The experimental results of the proposed algorithm are presented and discussed in Section 3. Section 4 draws the conclusions of this paper.

## 2. Materials and Methods

### 2.1. System Modeling

#### 2.1.1. Pedestrian Indoor Positioning System Model

**Figure 1:**
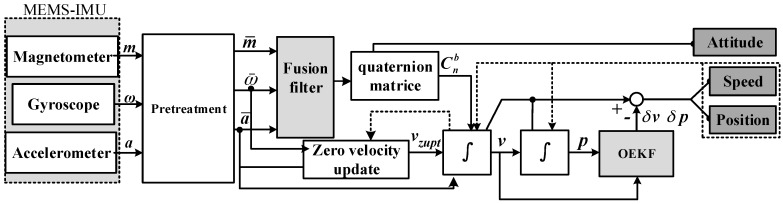
Pedestrian indoor positioning system model.

The pedestrian indoor positioning system model is shown in Figure 1, which shows the whole process from the data acquisition to the output of position and attitude. The MEMS-IMU obtains information on acceleration, angular rate, and magnetic field intensity. The initial information is prefiltered to reduce measurement noise. Then, the information on angular rate and magnetic field intensity is used to determine the attitude through the quaternion method, and the information on angular rate and acceleration is used to determine the velocity and position through two integrals. The zero velocity intervals determined by the zero velocity update algorithm are used to improve the accuracy of the velocity. In OEKF, the errors of angular rate, acceleration, velocity, and position are taken as state vectors, and the updated velocity and position are taken as measurement vectors.

#### 2.1.2. Analysis of Pedestrian Kinematics

**Figure 2:**
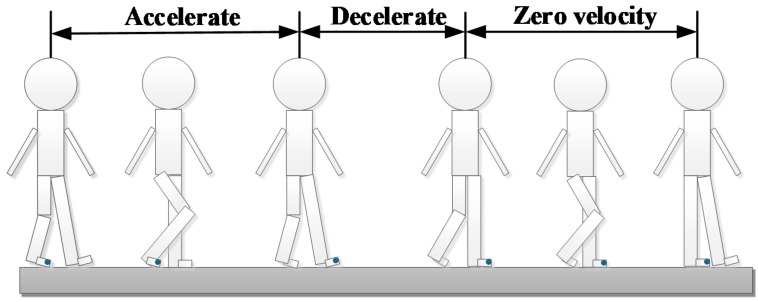
Analysis of pedestrian kinematics.

As the pedestrian walks, the left and right feet move alternately. Each stride can be modeled as a process of acceleration and deceleration. As shown in Figure 2, the foot accelerates as the heel leaves the ground and decelerates as the heel touches the ground again. A cycle of “acceleration–deceleration–zero velocity–acceleration–deceleration” occurs within each stride.

#### 2.1.3. Inertial Sensor Error Model

Affected by the manufacturing process and the application environment, a low signal-to-noise ratio limits the accuracy of the inertial navigation system. The errors in inertial sensors mainly consists of random errors. Analyzing random errors in inertial sensors is feasible for improving the accuracy of inertial navigation systems. The traditional methods of analyzing random errors include the power spectral density [36], autocorrelation analysis [37], and the Allan variance [38]. The Allan variance is widely used because it is able to distinguish different error sources and can be calculated and separated easily. However, the Allan variance suffers from energy leakage in constructing the error model [39] and low accuracy of estimation [40]. The wavelet decomposition algorithm can decompose random errors and reduce energy leakage effectively [41]. Wavelet variance can be obtained using the following equation:

(1) σx2(τ)=var{∑txtψ(t2τ−jτ)2τ}τ

where *x_t_* represents a sequence of *n* samples, *τ* = 1, 2, …, *n*/2 represents a scaling factor, *j* = 0, 2*τ*, 4*τ*, …, *n* − 2*τ* represents a time offset, and *ψ*(·) represents scaling and translation functions of the basic wavelet.

**Figure 3:**
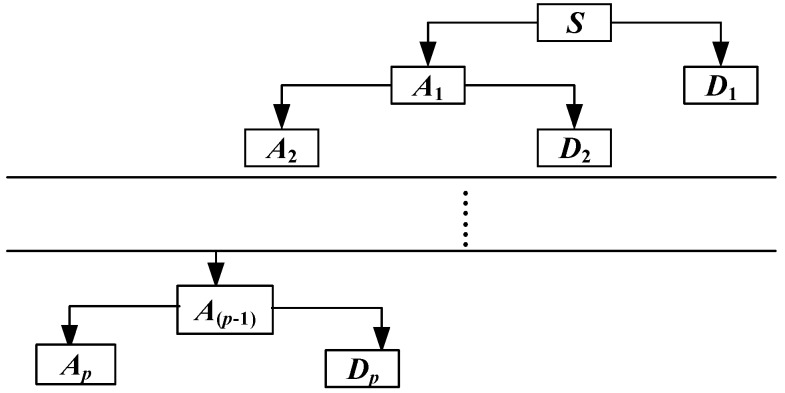
Wavelet decomposition algorithm.

The wavelet decomposition algorithm is used to process and reconstruct the inertial signal. The signal is decomposed into different components according to the frequency characteristics in the wavelet domain and is reconstructed without the random error. The wavelet decomposition algorithm is shown in Figure 3. The signal *S* is decomposed into one low-frequency and *p*-many high-frequency components by the wavelet decomposition:

(2) $$\mathrm{WT}(S)=A_{p}+\sum_{i=1}^{p}D_{i}$$

where ***A****_P_* represents the low-frequency component and ***D**_p_* represents the high-frequency components.

#### 2.1.4. Attitude Fusion Filter Algorithm

The accuracy of the attitude matrix plays a key role in inertial navigation systems, and directly affects the accuracy of attitude, velocity, and position. The gyroscope is vulnerable to static drift, so errors are accumulated when calculating attitude. On the other hand, the accelerometer and magnetometer have poor dynamic response, without accumulated errors. Therefore, the gyroscope, accelerometer and magnetometer can be used to complement each other where the accelerometer and magnetometer are used to determine attitude information under static conditions and the gyroscope is used to determine attitude information under dynamic conditions.

**Figure 4:**
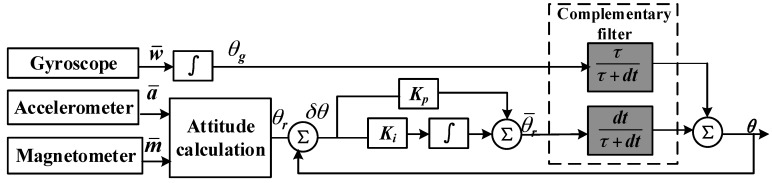
Attitude fusion filter algorithm.

The attitude fusion filter algorithm, shown in Figure 4, is used to improve the accuracy of the attitude. The principle is that the difference between the initial and final attitude angles is Proportional Integral (PI) controlled, then the balance filtering algorithm is used to fuse the attitude to improve the attitude accuracy and dynamic response.

In Figure 4, ***K****p* and ***K****i* are the proportional and integral coefficients in the PI controller and are used to decrease the errors in the attitude angle calculated by the accelerometer and the magnetometer. *∫* represents the integral operation, ττ+dt represents a high-pass filter, and dtτ+dt represents a low-pass filter. The accelerometer and magnetometer measurements are filtered through the low-pass filter to attenuate the high-frequency jitter in the attitude measurement; the gyroscope measurements are filtered through the high-pass filter to attenuate the accumulated drift errors.

The following equations can be obtained from Figure 4:

(3) $$\delta \theta =\theta_{r}-\theta$$

(4) $${\bar{\theta }}_{r}=K_{p}\delta \theta +\int_{0}^{t}K_{i}\delta \theta dt$$

(5) $$\theta =\frac{dt}{\tau +dt}{\bar{\theta }}_{r}+\frac{\tau }{\tau +dt}\theta_{a}$$

where *δθ* represents the error between the initial and final attitudes, *θr* represents the initial attitude calculated by the magnetometer and the accelerometer, ${\bar{\theta }}_{r}$ represents the updated attitude after the PI controller, and *θ* represents the attitude angle after fusing and filtering.

#### 2.1.5. Zero Velocity Update Algorithm

Based on the above analysis of pedestrian kinematics, there are two times during a movement cycle when the pedestrian’s feet are in complete contact with the ground; these are called zero velocity intervals. It is necessary to determine the zero velocity intervals accurately to improve the accuracy of the pedestrian inertial navigation and positioning algorithm. When the pedestrian’s foot is in full contact with the ground, the angular rate and horizontal acceleration of the foot are approximately equal to zero, while the vertical acceleration is approximately equal to gravitational acceleration g. The information about the acceleration and angular rate is used to determine the zero velocity intervals of a pedestrian’s movement cycle [33,34,35]. This paper uses a multicondition threshold discriminant algorithm to determine the zero velocity intervals as follows:

(6) $$C_{1}(t)=\{\begin{array}{c} 1\\ 0 \end{array}\begin{array}{c} T_{a\_\min}<\left|a_{t}\right|<T_{a\_\max}\\ \mathrm{otherwise} \end{array}$$

(7) $$C_{2}(t)=\{\begin{array}{c} 1\\ 0 \end{array}\begin{array}{c} \sigma_{a_{t}}^{2}<T_{a\_\sigma_{a}}\\ \mathrm{otherwise} \end{array}$$

(8) $$C_{3}(t)=\{\begin{array}{c} 1\\ 0 \end{array}\begin{array}{c} \left|\omega_{t}\right|<T_{\omega \_\max}\\ \mathrm{otherwise} \end{array}$$

where |*a_t_*| and |*ω_t_*| are the amplitudes of acceleration and angular rate at time *t.* $\sigma_{a_{t}}^{2}$ represents the acceleration variance at time *t* and can be expressed as

(9) $$\sigma_{a}^{2}(t)=\frac{1}{n-1}\sum_{t=i}^{i+n-1}{\left(a_{t}-{\bar{a}}_{n}\right)}^{2}$$

where ${\bar{a}}_{n}$ is the average value of acceleration within the window and *n* is the width of the window.

According to the logical operation “and”, the results of (6)–(8) at time *t* are processed; that is, ZUPT(*t*) = *C*_1_(*t*) & *C*_2_(*t*) & *C*_3_(*t*), and the zero velocity intervals are accurately determined.

If the zero velocity state is detected, the acceleration errors *δa_t_* should be reset. This is done as follows:

(10) $$\delta a_{t}={\left[\begin{array}{ccc} a_{x,k} & a_{y,k} & a_{z,k} \end{array}\right]}^{T}-{\left[\begin{array}{ccc} 0 & 0 & g \end{array}\right]}^{T}$$

where *a_x_*_,_*_k_*, *a_y_*_,__k_, *a_z_*_,_*_k_* are the acceleration values at moment *k*.

### 2.2. The Optimal Enhanced Kalman Filter

Based on the simplified Sage–Husa adaptive filtering algorithm, an enhanced adaptive filter is proposed in which orthogonal Kalman filters, covariance matching techniques, and activation functions are used to improve the accuracy of pedestrian indoor inertial positioning systems. In the filter, the errors of angular rate, velocity, acceleration, and position are taken as state vectors, the velocity and position by calculation are taken as the measurement vectors.

Due to the fact that it is difficult to obtain the exact mathematical model of the system and the statistical properties of the noise, the accuracy of the Kalman filter is reduced and the filter diverges. The Sage–Husa adaptive filtering algorithm can estimate and correct the statistical characteristics of noise. However, the Sage–Husa adaptive filtering algorithm cannot precisely estimate both process noise *Q* and measurement noise *R*. It is generally considered that process noise in the pedestrian inertial positioning system is stable and only the measurement noise needs to be estimated. Assuming that *Q* is known, a simplified Sage–Husa adaptive filtering algorithm is used to estimate *R*. The specific algorithm is shown as follows:

(11) $${\hat{X}}_{k}={\hat{X}}_{k/k-1}+K_{k}v_{k}$$

(12) $${\hat{X}}_{k/k-1}=\Phi_{k/k-1}{\hat{X}}_{k-1}$$

(13) $$v_{k}=Z_{k}-H_{k}{\hat{X}}_{k/k-1}$$

(14) $$K_{k}=P_{k/k-1}H_{k}^{T}{\left[H_{k}P_{k/k-1}H_{k}^{T}+R_{k}\right]}^{-1}$$

(15) $$P_{k/k-1}=\Phi_{k/k-1}P_{k-1}\Phi_{k/k-1}^{T}+Q_{k}$$

(16) $$P_{k}=\left[I-K_{k}H_{k}\right]P_{k/k-1}{\left[I-K_{k}H_{k}\right]}^{T}+K_{k}R_{k-1}K_{k}^{T}$$

(17) $$R_{k}=\left(1-d_{k}\right)R_{k-1}+d_{k}\left\{\left[I-H_{k}K_{k-1}\right]v_{k}v_{k}^{T}{\left[I-H_{k}K_{k-1}\right]}^{T}+H_{k}P_{k-1}H_{k}^{T}\right\}$$

where *d_k_* = (1 − *b*)/(1 − *b^k+^*^1^) and *b* is the forgetting factor ranging from 0.95 to 0.99.

The simplified Sage–Husa adaptive filter needs to estimate the noise characteristics of each filter process. When there are problems with the positioning system such as high order, short sampling time, and increased calculations, the accuracy of the simplified Sage–Husa adaptive filter is reduced or even diverges.

In order to solve the above problems, we first judge whether the outliers exist in the measurement data according to orthogonality of innovation. Then, the activation function is used to suppress outliers, and the strong tracking filter is introduced to suppress filter divergence.

#### 2.2.1. Determining Outliers

Because innovation *v_k_* has orthogonality, the orthogonality of *v_k_* changes when outliers appear in the measurement data. Therefore, the orthogonality of *v_k_* is analyzed to detect outliers in the measurement values.

According to the orthogonality of innovation,

(18) $$E\left(Z_{k}Z_{k}^{T}\right)=H_{k}P_{k/k-1}H_{k}^{T}+R_{k}+H_{k}X_{k/k-1}X_{k/k-1}^{T}H_{k}^{T}$$

and we denote the right-hand side of Equation (18) as

(19) $$G_{k}=H_{k}P_{k/k-1}H_{k}^{T}+R_{k}+H_{k}X_{k/k-1}X_{k/k-1}^{T}H_{k}^{T}$$

From the diagonal elements of the matrices in (18), a judgement is made as to whether the component *Z_i,k_* of *Z_k_* is the outlier, and the discrimination is shown as follows:

(20) $$M_{i,k}\in \left[G_{i,k}-\varepsilon_{i},G_{i,k}+\varepsilon_{i}\right]$$

where *M_i,k_* and *G_i,k_* represent the *i*th diagonal element of $E\left(Z_{k}Z_{k}^{T}\right)$ and *G_k_*. If the above equation is valid, the measurement *Z_k_* is considered as the normal value, whether *Z_k_* is an outlier. Because the above equation has calculation errors, a disturbance value *ε_i_* is added.

After detecting the outliers, the activation function is used to weight *Z_k_* to exclude outliers and maintain the orthogonality of *v_k_*. The activation function is shown as follows:

(21) $$f_{i}=\{\begin{array}{cc} 1 & \sqrt{M_{i,k}}<\sqrt{G_{i,k}+\varepsilon_{i}}\\ \sqrt{\frac{G_{i,k}+\varepsilon_{i}}{M_{i,k}}} & \sqrt{M_{i,k}}\geq \sqrt{G_{i,k}+\varepsilon_{i}} \end{array}$$

If $\sqrt{M_{i,k}}<\sqrt{G_{i,k}+\varepsilon_{i}}$, then the weight value is a unit value, which does not change the sequence of innovation. If $\sqrt{M_{i,k}}\geq \sqrt{G_{i,k}+\varepsilon_{i}}$, then $\sqrt{G_{i,k}+\varepsilon_{i}/M_{i,k}}$, which is less than 1, is used as the weight to maintain the orthogonality of *v_k_*.

Note that if *ε_i_* is too large, some outliers may go undetected. Conversely, if *ε_i_* is too small, the false detection of outliers may occur. In practical applications, *ε_i_* needs to be determined according to the requirements of the application and the required accuracy of the measurement values.

#### 2.2.2. Determining Filter Divergence Using a Covariance Matching Algorithm

The covariance matching algorithm checks residuals and determines whether they are convergent. The criterion for determining filter convergence is

(22) $$v^{T}(k)v(k)>\lambda tr(E[v(k)v^{T}(k)])$$

where *λ* is the reserve coefficient and *tr* is the trace of the matrix. When *λ* > 1, the actual error exceeds the expected value and the filter has diverged.

The strong tracking filter has advantages of strong robustness against model uncertainty, strong tracking capability, and low calculation requirements. When the simplified Suge–Husa adaptive filter diverges, a strong tracking filter can be used to prevent the filter from diverging and to achieve good tracking performance in an environment with a low signal-to-noise ratio.

The strong tracking filter adopts a time-varying fading factor to fade the previous data, and adjusts the predictive error covariance matrix and the corresponding gain matrix in real time so that the residual sequences are always orthogonal to each other:

(23) $$E\left[v_{k+j}v_{k}^{T}\right]=0$$

where *k* = 0, 1, 2, …, *j* = 1, 2, …. The fading factor *μ_k_* is introduced to adjust the prediction covariance matrix *P_k_*_,*k*−1_:

(24) $$P_{k,k-1}=\mu_{k}\Phi_{k/k-1}P_{k-1}\Phi_{k/k-1}^{T}+Q_{k}.$$

The fading factor *μ_k_* can be determined using the following equations:

(25) $$\mu_{k}=\{\begin{array}{cc} \frac{tr(W_{k})}{tr(N_{k})} & \frac{tr(W_{k})}{tr(N_{k})}\geq 1\\ 1 & \frac{tr(W_{k})}{tr(N_{k})}<1 \end{array}$$

(26) $$W_{k}=v_{k}v_{k}^{T}-\Phi_{k}Q_{k}\Phi_{k}^{T}-R_{k}$$

(27) $$N_{k}=\Phi_{k}F_{k/k-1}P_{k/k-1}F_{k/k-1}^{T}\Phi_{k}^{T}.$$

**Figure 5:**
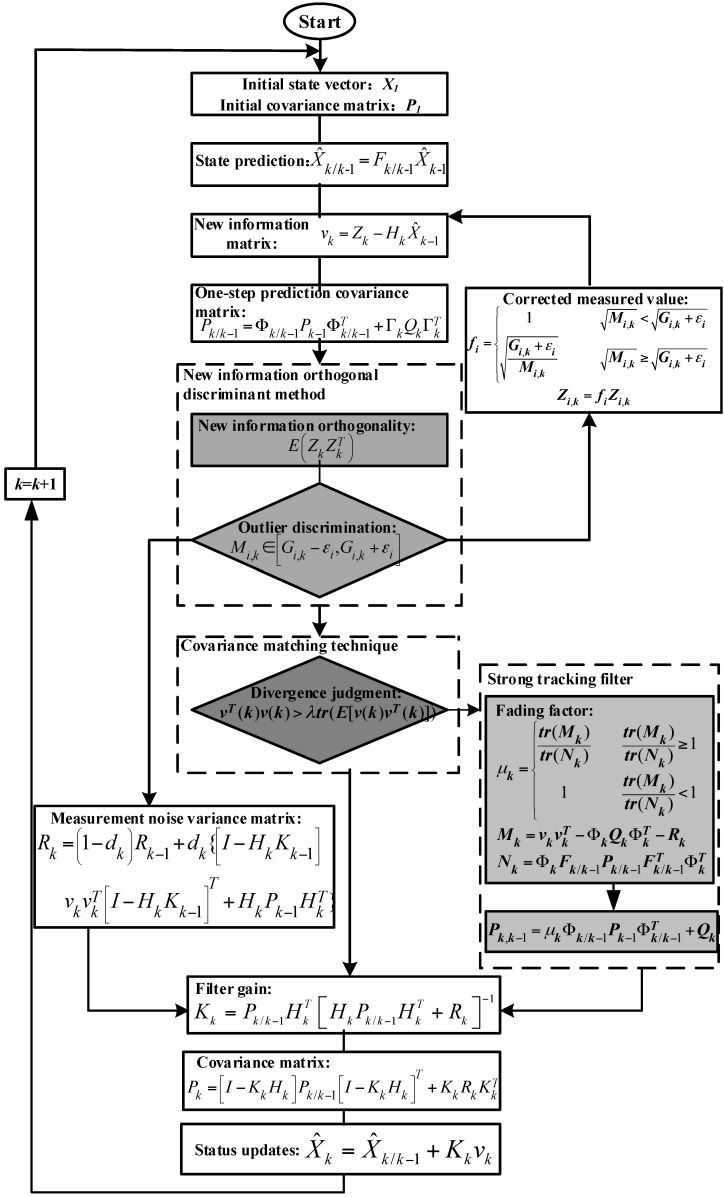
OEKF algorithm.

Figure 5 shows an optimal enhanced Kalman filter algorithm. Firstly, the initial state, covariance matrix, and the innovation matrix are set. Then, the innovation orthogonal discriminant is used to determine whether outliers exist in the measurement values. If the measurement values have outliers, then the measurement values are filtered. If the filter is divergent, a strong tracking filter is introduced to suppress the divergence.

## 3. Results

### 3.1. Experimental Device and Data Acquisition

To assess the performance of the pedestrian inertial navigation system in this paper, a micro-inertial navigation module was used. The data refresh rate of the micro-inertial navigation module was 100 Hz and a 32-bit ARMCortexM3 Microcontroller Unit (MCU) was used for calculations. Specific parameters are shown in Table 1.

**Figure 6:**
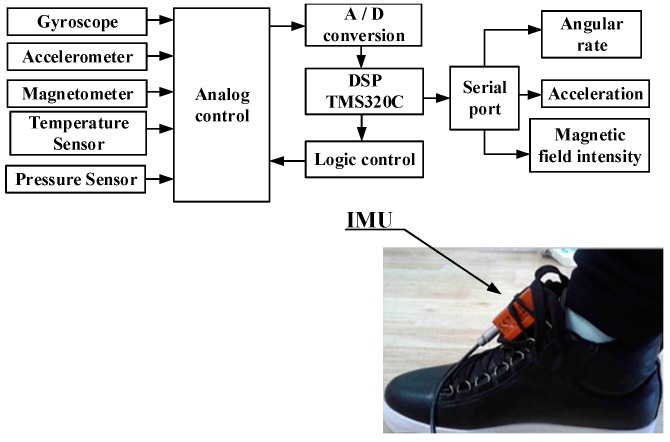
IMU structure and installation diagram.

The structure and installation of the pedestrian inertial navigation system are shown in Figure 6. The accelerometer, gyroscope, magnetometer, and other sensors were mounted on the Inter-Integrated Circuit(I2C) bus and data were transmitted from the serial port to the host computer through the Digital Signal Processing (DSP). The inertial navigation module was tied on the foot to obtain pedestrian movement information.

### 3.2. Experimental Environment Settings

**Figure 7:**
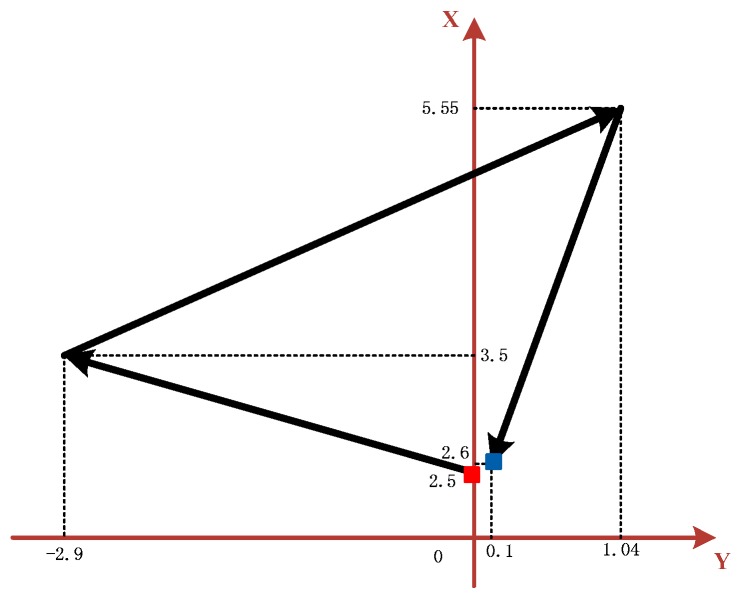
Pedestrian walking route.

The pedestrian walking route is shown in Figure 7. The red square represents the starting point, the blue square represents the ending point, and the arrow represents the walking direction. It is clear that the pedestrian starts from point (0, 2.5) and traverses through points (−2.9, 3.5) and (1.04, 5.55), eventually stopping at point (0.1, 2.6).

### 3.3. Analysis of Experiments

#### 3.3.1. Analysis of Errors of Inertial Sensor

**Figure 8:**
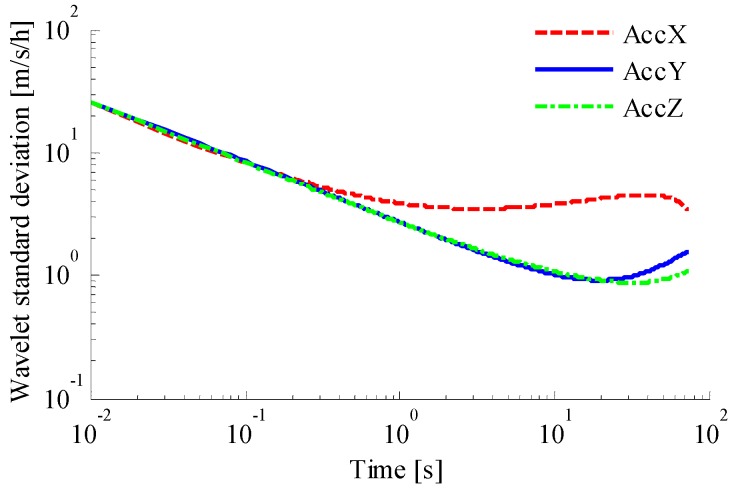
Analysis of accelerometer with variance.

**Figure 9:**
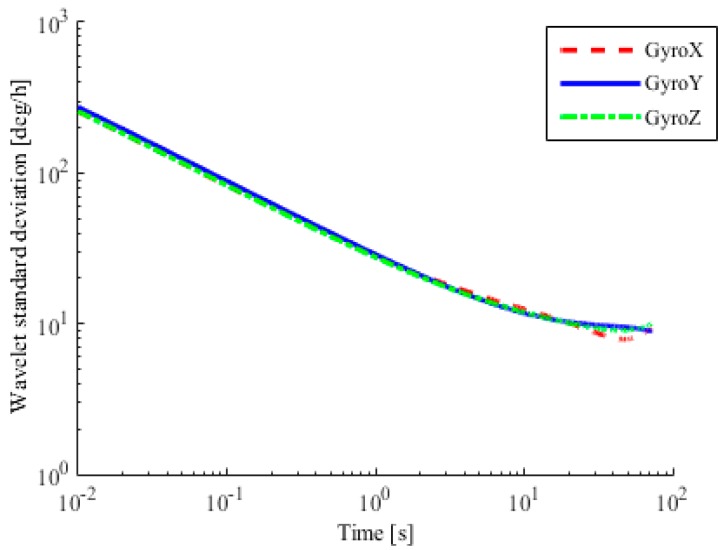
Analysis of gyroscope with variance.

To analyze the error sources of the accelerometer and gyroscope, the wavelet variance method is used, as shown in Figure 8 and Figure 9. It can be seen that the output value of the accelerometer is affected by acceleration random walk, instability of bias, velocity random walk, and quantization noise. The output value of the gyroscope is affected by angle random walk, bias instabilities, and quantization noise. Results of the wavelet analysis of variance are shown in Table 2 and Table 3. It can be seen that random noise in the accelerometer and gyroscope measurements consists of white noise and colored noise, before and after 10 s, respectively.

**Figure 10:**
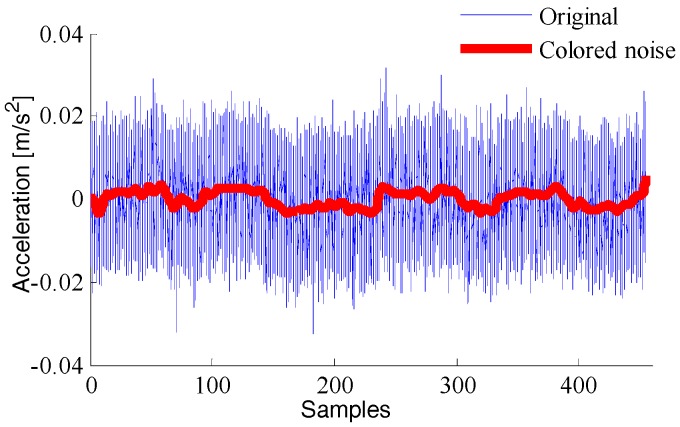
Comparison chart of wavelet decomposition.

**Figure 11:**
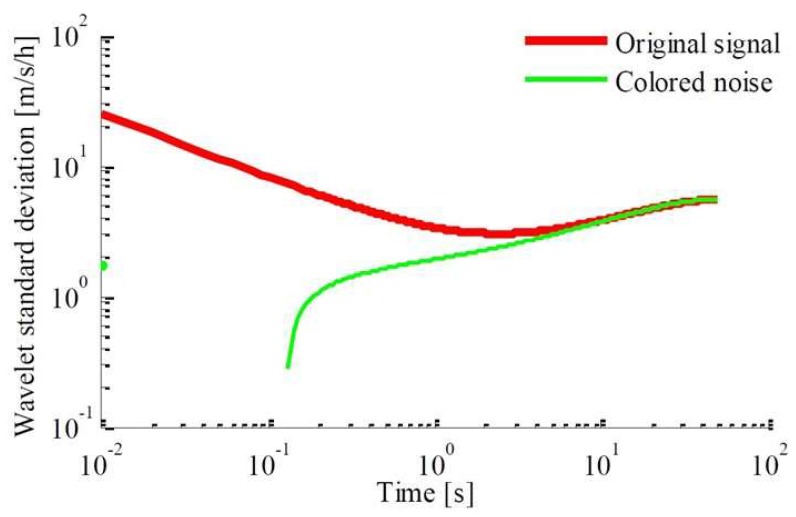
Comparison chart of decomposed signal in wavelet variance.

The wavelet decomposition algorithm is used to reconstruct the output value of the inertial sensor. Taking the output value of acceleration as an example, the “*db*6” wavelet basis function, threshold criterion of “*rigrsure*”, and soft threshold method are used to obtain the comparison between the original signal and the colored noise and the wavelet variance comparison between them, respectively, as shown in Figure 10 and Figure 11.

#### 3.3.2. Experimental Analysis of Attitude Information

**Figure 12:**
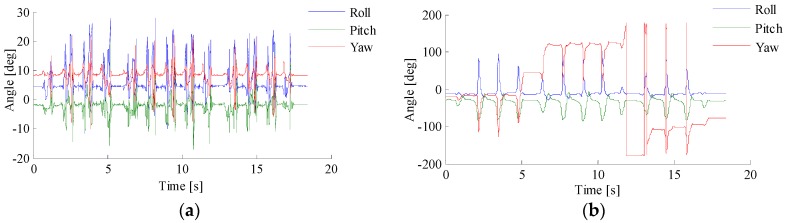
Comparison chart of gestures. (**a**) The initial attitude measurement; (**b**) The output of the attitude fusion filter.

The initial attitude measurement is shown in Figure 12a. It can be seen that there are measurement noise and cumulative errors in the measurement values. The output of the attitude fusion filter is shown in Figure 12b. It can be seen that the measurement noise and cumulative errors are attenuated, the output is free of glitches, and the accuracy of the attitude is improved.

#### 3.3.3. Experimental Analysis of Zero Velocity Update

**Figure 13:**
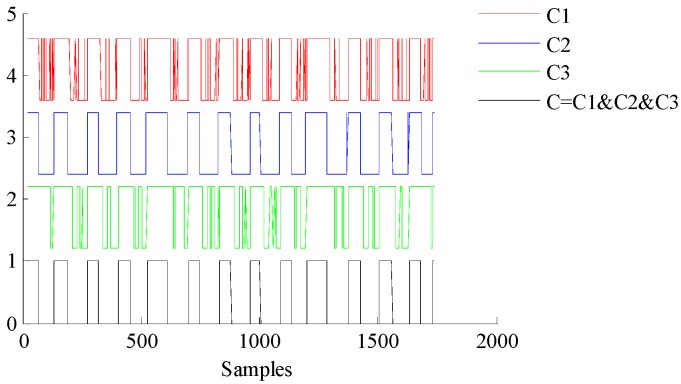
Acquisition of zero velocity interval.

**Figure 14:**
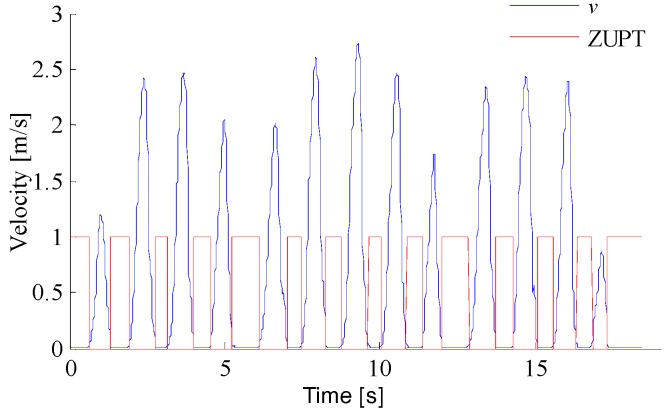
Velocity corrections in the Zero Velocity Update algorithm (ZUPT).

As shown in Figure 13, according to the outputs of the accelerometer, magnetometer, and gyroscope, the pedestrian zero velocity interval is obtained. The velocity of the pedestrian is corrected based on the zero velocity intervals, as shown in Figure 14. It can be seen that the cumulative errors in velocity and position decrease.

#### 3.3.4. Analysis of Different Positioning Systems

**Figure 15:**
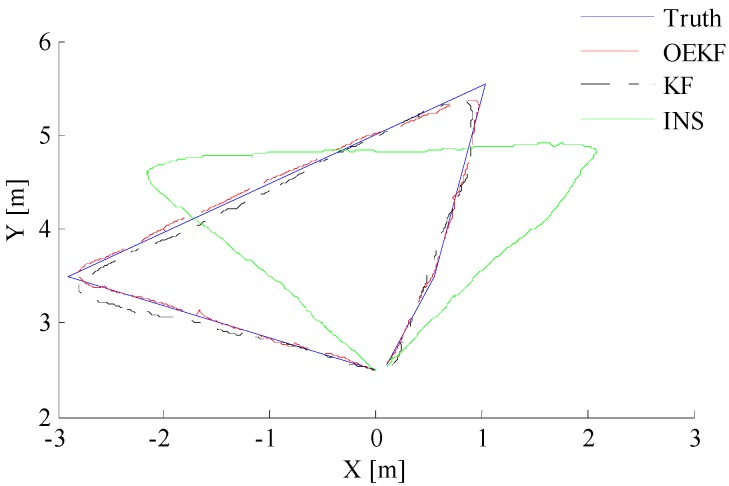
Comparison of trajectory under different positioning systems.

**Figure 16:**
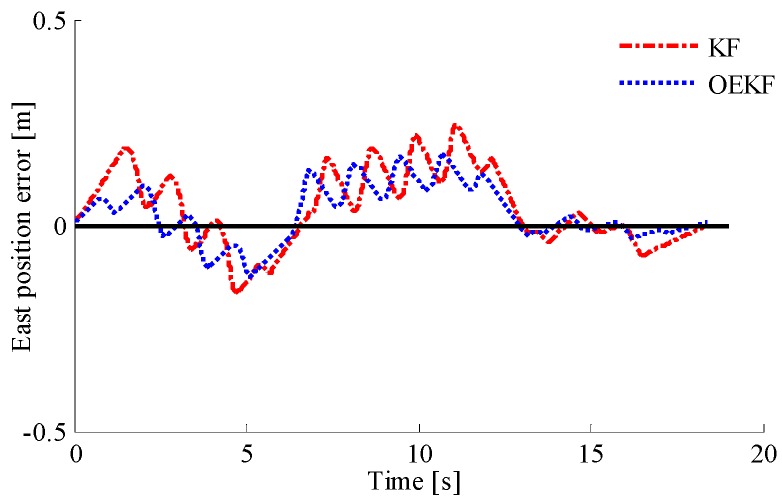
Comparison of east position errors.

**Figure 17:**
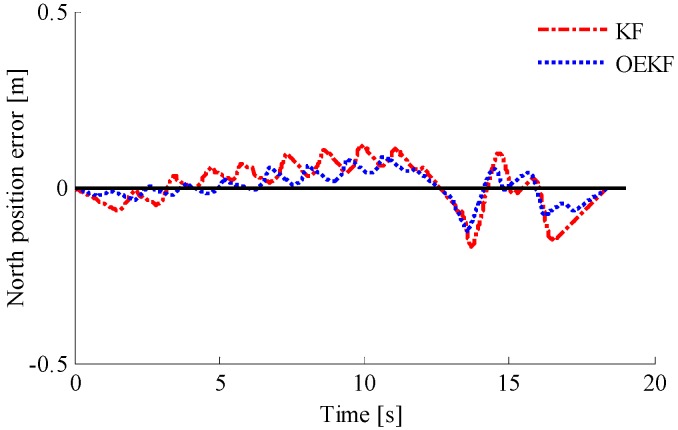
Comparison of north position errors.

In order to verify the performance of the optimal enhanced Kalman filtering algorithm, the positioning of pedestrians using different filtering algorithms is shown in Figure 15. It can be seen that the INS system directly using the outputs of accelerometer, gyroscope, and magnetometer has cumulative errors, showing a positioning track far away from the actual track. By contrast, the KF and OEKF algorithms eliminate the accumulated errors, and the performance of OEKF is better. Specific position errors in east and north directions are shown in Figure 16 and Figure 17. The figures show that the filtering effect of OEKF is better, and the cumulative errors in position are effectively offset.

The comparison between OEKF and KF is shown in Table 4. It can be seen that the east position error using KF is −0.1847 m to 0.2455 m, the root mean square error is 0.66 m, and the confidence is 97.144%. The east position error using OEKF is -0.1241m to 0.1738 m, the root mean square error is 0.0987 m, and the confidence level is increased by 1.3309%. The north position error using KF is −0.1688 m to 0.1222 m, the root mean square error is 0.0816 m, and the confidence level is 97.1855%. The north position error using OEKF is −0.1251 m to 0.0879 m, the root mean square error is 0.0360 m, and the confidence level is increased by 1.2522%.

## 4. Conclusions

The traditional inertial navigation system has problems such as low signal-to-noise ratio, cumulative errors, and outlier interference, and therefore cannot meet the requirements of accuracy in pedestrian positioning systems. To address these problems, an inertial positioning system based on OEKF is proposed in his paper. Firstly, wavelet decomposition is used to filter the inertial signal effectively. Then, the zero velocity update algorithm and the attitude fusion algorithm are used to suppress the accumulative errors of speed and attitude. Finally, the OEKF algorithm is proposed and compared with the INS and Kalman filter. The experimental results show that the OEKF filter algorithm is suitable for a pedestrian inertial positioning system, and effectively improves the pedestrian positioning accuracy. Further work will be done to study the impact of process noise on the positioning system, and process noise more accurate than a fixed value should be incorporated into the system.

## Figures and Tables

**Table 1:** MEMS-IMU Performance Parameters.

Senor	Standard Full Range	Noise Density	Band Width	Voltage
Accelerometer	50 m/s^2^	80 μg/√Hz	375 Hz	4.5 V
Gyroscope	450°/s	0.01°/s/√Hz	450 Hz	4.5 V
Magnetometer	±80 μT	200 μG/√Hz	N/A	4.5 V

**Table 2:** Analysis of accelerometer with wavelet variance.

Error Item	AccX	AccY	AccZ
Acceleration random walk m/s/h^3/2^	80.953	7.8644	6.5712
Instability of bias m/s/h	4.3845	0.73242	1.0858
Velocity random walk m/s/h^1/2^	0.040361	0.044576	0.043489
Quantization noise m/s	0.035239	0.040676	0.040221

**Table 3:** Analysis of gyroscope with wavelet variance.

Error Item	GyroX	GyroY	GyroZ
Angle random walk °/h^1/2^	0.43234	0.46484	0.43525
Instability of bias °/h	16.296	10.872	13.737
Quantization noise μrad	1.2229	1.5065	1.3845

**Table 4:** Comparison of error in different trajectory positions.

	KF		OEKF	
	East	North	East	North
Range of error (m)	−0.1847 to 0.2455	−0.1688 to 0.1222	−0.1241to 0.1738	−0.1251 to 0.0879
Root mean square error (m)	0.66	0.0816	0.0987	0.0360
Residual rate (%)	2.8560	2.8145	1.5251	1.5623
Confidence (%)	97.144	97.1855	98.4749	98.4377
